# Evaluation of Nosocomial Infection Control Programs in health services^1^

**DOI:** 10.1590/0104-1169.0113.2530

**Published:** 2015

**Authors:** Mayra Gonçalves Menegueti, Silvia Rita Marin da Silva Canini, Fernando Bellissimo-Rodrigues, Ana Maria Laus

**Affiliations:** 2 MSc, RN, Hospital das Clínicas, Faculdade de Medicina de Ribeirão Preto, Universidade de São Paulo, Ribeirão Preto, SP, Brazil; 3 PhD, Associate Professor, Escola de Enfermagem de Ribeirão Preto, Universidade de São Paulo, WHO Collaborating Centre for Nursing Research Development, Ribeirão Preto, SP, Brazil; 4 PhD, Professor, Faculdade de Medicina de Ribeirão Preto, Universidade de São Paulo, Ribeirão Preto, SP, Brazil

**Keywords:** Indicators of Health Services, Health Evaluation, Hospital Infection Control Program

## Abstract

**OBJECTIVES:**

to evaluate the Nosocomial Infection Control Programs in hospital institutions regarding structure and process indicators.

**METHOD:**

this is a descriptive, exploratory and quantitative study conducted in 2013. The study population comprised 13 Nosocomial Infection Control Programs of health services in a Brazilian city of the state of São Paulo. Public domain instruments available in the Manual of Evaluation Indicators of Nosocomial Infection Control Practices were used.

**RESULTS:**

The indicators with the highest average compliance were "Evaluation of the Structure of the Nosocomial Infection Control Programs" (75%) and "Evaluation of the Epidemiological Surveillance System of Nosocomial Infection" (82%) and those with the lowest mean compliance scores were "Evaluation of Operational Guidelines" (58.97%) and "Evaluation of Activities of Control and Prevention of Nosocomial Infection" (60.29%).

**CONCLUSION:**

The use of indicators identified that, despite having produced knowledge about prevention and control of nosocomial infections, there is still a large gap between the practice and the recommendations.

## Introduction

According to the Ministry of Health^(^ 1 ^)^, nosocomial infection is the infection acquired after the client's admission to hospital and manifested during hospitalization or after discharge, provided that it can be related to hospitalization or hospital procedures. Since infections are not limited to the hospital environment, the terminology Healthcare-Associated Infection has been considered more appropriate.

Nosocomial infections (NI) are of great epidemiological relevance by raising the morbidity and mortality rates, extending the length of stay of patients in hospital and thus burdening the cost of treatment^(^ 2 ^-^ 3 ^)^. 

Estimates of developed countries indicate that at least 5% of patients in hospitals acquire infection^(^ 4 ^)^. In Brazil, research^(^ 5 ^)^ undertaken in 2009 at a university hospital found an average annual prevalence rate of NI of 8.2%, and 149 (29.1%) cases of pneumonia, 136 (26.6%) bloodstream infections, 87 (17%) urinary tract infections, 57 (11.1%) central catheter infections and 47 (9.2%) surgical site infections.

Constant monitoring of health practices should focus on costs and quality for patient safety. The use of clinical indicators, defined as continuous or periodic quantitative measures of variables, characteristics or attributes of a given process or system, are becoming a useful tool for assessing the health services^(^ 6 ^)^. 

Although there is national legislation recommending the establishment of nosocomial infection control programs (NICPs) in health facilities, the current evaluation system does not favor the measuring, interpretation and qualification of the evaluation, which should be considered insufficient to determine the quality of care practices^(^ 7 ^)^. In this context, it was considered appropriate to carry out the present study, which aimed to evaluate the structure and process indicators of NICPs in hospitals of the city of Ribeirão Preto.

## Methods

This is a descriptive and exploratory study with a quantitative approach, performed in the city of Ribeirão Preto, in 2013. The Nosocomial Infection Control Committees of health services were identified by using the National Health Facilities Cadaster (NHFC) for reference, which categorizes the hospitals as public, private and non-profit, general or specialized. Health services for treatment of mental illness were excluded. 

In the data collection period, the city of Ribeirão Preto had 16 NICPs. The population consisted of 13 NICPs (81.25%), due to the refusal of three institutions to participate.

Data were obtained through interviews with members of the Nosocomial Infection Control Committee (NICC) of the participant institutions and analysis of documents in order to identify the practices that compose each of the indicators evaluated. 

The instruments used in the form of procedural clinical indicators, previously constructed and validated^(^ 7 ^)^ regarding their content, are available in the Manual of Evaluation Indicators of Nosocomial Infection Control Practices in the public domain. 

For the calculation of quality compliance rates of the health service NICPs of, formulas recommended in operational constructs of these indicators were used through their arrangement as numerators and denominators. Denominators always correspond to the total evaluated practices and the numerators to the total practices that obtained compliance. 

Two criteria were also considered: 1) partial compliance of quality of NICPs, when institutions did not fully meet the indicator requirements, such as: presentation of NI prevention manuals used by the institution, outdated, or even only part of the documentation required; 2) "does not apply" when the institution did not have the service, the area or the type of care that was being evaluated.

Data were entered and stored in a database and analyzed using EpiInfo, version 6. The project was approved by the Ethics Committee of the University of Sao Paulo at Ribeirão Preto College of Nursing (CAAE), under protocol number 02889412.2.0000.5393.

## Results

Most of the hospitals, nine (69.23%) fell into the category of general hospital, seven (53.84%) were private institutions with up to 70 beds and four (30%) had accreditation. 

The NICC was constituted in 100% of the institutions and in almost half of services (46.16%) for over ten years. Among the NICC professionals, 23 (69.69%) had less than five years of experience.

It is noteworthy that all 33 (100%) professionals surveyed reported that they did not receive specific training to operate in this service or had expertise in the area. Specifically, for the category of nurses, 12 (57%) did not have prior experience and expertise in the area, while all members of the medical team 12 (100%) reported that they had performed medical residency in infectious diseases. 

When analyzing the Indicator "Evaluation of technical and operational structure of the NICP", the average compliance of institutions was 75%, six programs had 100% compliance in the items and only one had 20% (Table 1).

**Table 1 -:** Compliance values by item of the Indicator "Evaluation of Technical and Operational Structure of the Nosocomial Infection Control and Prevention Program" applied to health facilities. Ribeirão Preto, SP, Brazil, 2013

Indicator 1		Compliance			Non-compliance			Partial compliance			Does not apply
		n	%		n	%		n	%		n
Component											
	The Commission is represented, at least, by members of the medical service, nursing and administration.	12	92.31		1	7.69		0			0
	There is a charter that determines the functioning of the Commission.	12	92.31		0			1	7.69		0
	There are two health professionals with higher education performing actions of prevention and control of infection, for every 200 beds, one of whom is a nurse.	8	61.54		5	38.46		0			0
	The nurse acts exclusively dedicated to the service, at least 6 hours a day.	8	61.54		5	38.46		0			0
	There are other professionals, with higher education, who act exclusively dedicated to the service, at least 4 hours a day.	7	53.85		6	46.15		0			0
	The Commission holds regular meetings with participation of members and leaders.	10	76.92		1	7.69		2	15.39		0
	There is support of own or outsourced microbiology and pathology laboratories.	13	100.00		0			0			0
	There is physical space limited and exclusive for daily activities, archives etc. of the Commission.	8	61.54		4	30.77		1	7.69		0
	There is availability of computer resources for the activities of the Commission.	12	92.31		1	7.69		0			0
	The administration provides statistical data (number of admissions, discharges, deaths, patients-day etc.) to carry out the Commission’s reports.	10	76.92		3	23.08		0			0
Mean		75.38			20.00			4.62			-

Three components of the indicator were considered in partial compliance, one was related to the bylaws, that is, an institution declared to have them but did not present them during the interview and evaluation; another one related to the physical space had a defined area, but it was not exclusive to the service and to the conduction of regular meetings. The institutions presented minutes, but with the date of the year before.

The Indicator "Evaluation of the Operational Guidelines for the Control and Prevention NI" had a compliance average of 58.97%, but only one institution reached 100%, and another one reached 6.67%. 

The hospitals that presented written routine and manuals, although outdated, i.e., prepared two years before or more were categorized as partial compliance. The evaluation of this indicator as specialized services, with day-hospital inpatients, had some components that did not apply to this process (Table 2).

**Table 2 -:** Compliance values by item of the Indicator "Evaluation of the Operational Guideline of the Nosocomial Infection Control and Prevention Program" applied to health facilities. Ribeirão Preto, SP, Brazil, 2013

Indicator 2		Compliance			Non-compliance			Partial compliance			Does not apply
		n	%		n	%		n	%		n
Component											
	There is a recommendation for assessment and referral of injuries caused by sharps and biological material.	9	69.23		1	7.69		3	23.08		0
	There are recommendations for waste disposal.	8	61.54		0			5	38.46		0
	There are recommendations for control and prevention of respiratory infections.	7	58.33			33.33		1	8.34		1
	There are recommendations for control and prevention of urinary tract infections.	7	58.33		3	25		2	16.67		1
	There are recommendations for control and prevention of bloodstream infections.	8	61.54		5	38.46		0			0
	There are recommendations for control and prevention of surgical site infections.	8	61.54		4	30.77		1	7.69		0
	There are recommendations for isolation of patients with infectious and contagious diseases.	8	66.67		3	25		1	8.33		1
	There is recommendation for the use of prophylactic antibiotics.	9	69.24		2	15.38		2	15.38		0
	There is standardization of germicidal and antiseptic solutions.	7	53.85		6	46.15		0			0
	There is a recommendation for cleaning, disinfection and sterilization techniques of materials.	8	61.54		3	23.08		2	15.38		0
	There is a recommendation for hand hygiene technique.	9	69.24		2	15.38		2	15.38		0
	There is a recommendation for cleaning and disinfecting surfaces.	9	69.23		3	23.08		1	7.69		0
	There is a recommendation for washing and sanitizing clothes used in the institution.	4	30.77		5	38.46		4	30.77		0
	There is a technical recommendation for collecting material to perform cultures.	6	46.16		5	38.46		2	15.38		0
	There is a recommendation for bandage techniques and frequency of their change.	7	53.85		4	30.77		2	15.38		0
Mean		59.40			26.07			14.53			-

The Indicator "Evaluation of the Epidemiological Surveillance System of NI" had an average compliance of 82%, six programs had 100% of compliance items and only one had 11% (Table 3).

**Table 3 -:** Compliance values by item of the Indicator "Evaluation of the Epidemiological Surveillance System of Nosocomial Infection" applied to health facilities. Ribeirão Preto, SP, Brazil, 2013

Indicator 3		Compliance			Non- compliance			Partial Compliance			Does not apply
		n	%		n	%		N	%		n
Component											
	Conducts epidemiological surveillance at fixed intervals.	12	92.31		1	7.69		0			0
	Conducts surveillance of nosocomial infection through active case search.	12	92.31		1	7.69		0			0
	Conducts active search of nosocomial infection in high-risk units.	8	100		0.00			0			5
	Monitors with regular intervals and records the microbiological culture results, which identify resistant microorganism’s strains or species.	10	83.33		2	16.67		0			1
	There are predetermined criteria for diagnosis of nosocomial infection.	8	61.54		3	23.08		2	15.38		0
	Produces periodic report of results of epidemiological surveillance.	12	92.31		1	7.69		0			0
	The reports examine and report changes in the epidemiological profile.	12	92.31		1	7.69		0			0
	The reports correlate results with control and prevention strategies adopted.	6	46.15		6	46.16		1	7.69		0
	The reports are regularly available to sectors and leaders.	10	76.92		3	23.08		0			0
	The reports are regularly available to public bodies.	100	100		0			0			0
	Mean	83.72			13.97			2.31			-

The situations where there was no evidence of correlation of the results of NI search with control and prevention strategies were considered as partial compliance, as well as those where the NICC did not used predetermined criteria (according to the literature) for the diagnosis of all nosocomial infections. Five NICPs had the item active search for cases of NI in high-risk units assessed as does not apply, because of the lack of these units. In addition, one had the same assessment of the item regarding the frequent monitoring of microbiological culture results, since the patients' profile and the type of activities carried out did not require such monitoring.

The Indicator "Evaluation of Control and Prevention Activities of NI" had an average compliance of 60.29%. It is highlighted that six institutions achieved 100% of compliance in the items and one did not presented compliance in any item (Table 4).

**Table 4 -:** Compliance values by item of the Indicator "Evaluation of Control and Prevention Activities of Nosocomial Infection" applied to health facilities. Ribeirão Preto, SP, Brazil, 2013

Indicator 4		Compliance			Non- compliance			Partial compliance			Non- compliance
		n	%		n	%		n	%		n
Component											
	Dialysis unit	4	66.68		1	16.66		1	16.66		7
	Blood bank	1	20		3	60.00		1	20		8
	Clinical analysis laboratory	0			5	83.33		1	16.67		7
	Pathological anatomy laboratory	0			4	80		1	20		8
	Inpatient units	12	92.31		1	7.69		0			0
	Intensive care units	8	100.00		0			0			5
	Baby nursery	5	100.00		0			0			8
	Center of material and sterilization	10	76.93		1	7.69		2	15.38		0
	Surgical center	10	76.93		1	7.69		2	15.38		0
	Emergency room	3	50		2	33.33		1	16.67		7
	Ambulatory	5	55.56		1	11.11		3	33.33		4
	Nutrition and dietetics service	8	61.54		3	23.08		2	15.38		0
	Participates in the technical decisions for product specification and purchase	5	38.46		8	61.54		0			0
Mean		56.80			30.16			13.04			-

The NICCs that reported performing inspection, orientation upon spontaneous demand or assessment according to specific legislation or hospital policy in specific units and facility visits were considered as partial compliance since they did not have evidence reports of these activities. In cases where the institution did not have the specialized service, it was considered in the does not apply category.

## Discussion

There was adherence of 81.2% of the institutions in Ribeirão Preto to this investigation. A similar survey conducted in the city of São Paulo had a participation percentage of 31%^(^ 7 ^)^. Thus, it was considered that the results reflect the diagnosis of NICP in this city.

The best performance was obtained in the Indicators "Evaluation of technical and operational structure" and "Evaluation of the epidemiological surveillance system." It is highlighted that, in the first indicator, most items encompassed national legal requirements, including human resources for composition of NICCs, physical space and implementing activities^(^ 1 ^)^, which explains the high compliance rate.

Research conducted in Ontario, Canada, showed a deficit of hours/professionals for activities for the control of nosocomial infection per 100 beds, providing evidence that this ratio was appropriate in only 22.6%^(^ 8 ^)^.

On the other hand, when analyzing the development and updating of manuals of rules, routines and recommendations for control of NI, it was observed that there has been compliance in more than half of the institutions (59.4%), although much of the NICCs do not meet this minimum requirement for realization of safe care, which also constitutes a legal requirement.

Effective programs should meet the minimum established by law and still have, in its scope, actions such as collection system, management, analysis and reporting of data with a continuous improvement plan; formal policies and procedures; study programs, education and training^(^ 9 ^)^.

For the indicator "Evaluation of the epidemiological surveillance system", the items include forms of surveillance, diagnostic criteria of NI and preparation of technical and scientific reports. Although the indicator has reached a high compliance rate, it was observed that the items "existence of predetermined criteria for diagnosis of infection" and "correlation of results with control and prevention strategies" had the highest rates of non-compliance. Deviating data were highlighted in a study conducted in São Paulo, but it should also be considered that all participating institutions were accredited^(^ 7 ^)^.

Another component with low compliance was related to the criteria used for the diagnosis of NI and, on average, only 60% of institutions performed it. The lack of these may not reflect the reality of nosocomial infection incidence rates that may be over- or underestimated and consequently prejudice the implementation of control and prevention actions. A study conducted in 10 European countries, also identified disagreement in the notification criteria. Twelve reports of suspected cases of surgical site infection were subject to 100 infectologists who work in infection control and 86 surgeons. It was observed that the professionals disagreed regarding the diagnosis of infection and there was also variation in different countries^(^ 10 ^)^.

For the indicator "Evaluation of operational guidelines", the indices with lower compliance were related to standardization of germicidal and antiseptic solutions, recommendation for prevention and control of infection of the bloodstream, technical recommendation for collection of material for culture and recommendation for washing and cleaning of clothes. 

The recommendation for washing and cleaning of clothes was in compliance in 30.7% of institutions. A similar research has found that this item had lower compliance rate, but with a much higher average (64%)^(^ 7 ^)^. The institutions that used outsourced laundry services, but had manuals with specific recommendations for the care of the clothes were considered in compliance.

Considering the item regarding the collection of materials for culture, it is imperative that institutions have specific recommendations, since the lack of technique standardization may compromise the identification of microorganisms related to infection and consequently generate unnecessary or incorrect treatment, burdening the care costs.

Regarding the indicator "Evaluation of activities of control and prevention of nosocomial infections", items that had worse results were related to participation in technical decisions for specifying and purchasing products, and blood bank and clinical analysis laboratory units.

It was observed that the NICCs of the participating institutions had lower compliance rate in activities related to the blood bank. It is known that, although many advances have occurred in recent decades regarding the transmission of Human Immunodeficiency Virus (HIV) and hepatitis viruses B and C, new security actions for quality control must be implemented due to epidemiological changes of transfusion-transmitted infections, which require studies with robust design to provide strong scientific evidences^(^ 11 ^)^.

Another item with low compliance rate was the participation in technical decisions for specification and purchase of consumer products (38.46%). The material management comprises a complex process involving costs and quality of care. Thus, with increasingly tight budgets, it is vital to adopt technical criteria to justify the selection and acquisition of materials without compromising professionals and customers safety^(^ 12 ^)^.

For an NICP to be considered as performing well, it is necessary that the indicators are in compliance. Thus, it is necessary to propose preventive measures, implementation and documentation of audits in the units of health institutions, analysis of problems through root cause identification, measurement of NI rates, return thereof to the professional units and comparison of these rates with other institutions at the local, state and national level. In addition, is necessary to monitor the results and provide feedback of data to the team^(^ 13 ^-^ 15 ^)^.

Well-structured programs can reduce nosocomial infection rates and, consequently, the economic burden of these events^(^ 16 ^)^.

 The work of experienced professionals with expertise in this subject has a substantial impact on the quality of this service. It is noteworthy that Brazil has a lack of specialized courses in NI control^(^ 17 ^)^, which points to the possibility of developing a new indicator to assess the technical training of professionals working in NICPs. This research has identified the lack of training for the early start of activity in this segment in 100% of the professionals, which supports the proposal for inclusion of this item in the evaluation process.

Although the existence of specific care in the institutions evaluated is recognized as the main limitation of this study, the results certainly enabled a comprehensive local assessment regarding the structure of NICPs. 

Based on the experience of the application of this indicator, which has been validated and used in previous research, it should be considered that, to carry out the assessment of NICPs, besides the documentation and reporting of NICC professionals, it is essential to observe the care practice services, with measurement of process indicators.

The possibility of further studies has been considered in order to map the Brazilian reality and support public policies to improve nosocomial infection control practices. It is recommended that the application of indicators for the evaluation of NICPs enter the routine evaluation in health services, including internal audits and health inspections.

The increase of research for the development of indicators that include specialized services, of low and medium complexity, would also be relevant in order to obtain reliable data to portray the reality of these institutions.

## Conclusion

The use of indicators identified that, despite having much knowledge produced on prevention and control of nosocomial infections, there is still a large gap between the practice and the recommendations.

This research identified that the evaluation indicators of the NICP are is feasible and can be used both by these programs and the units that carry out inspection in health institutions, as a tool to improve the activities carried out.
